# Prototype of biliary drug-eluting stent with photodynamic and chemotherapy using electrospinning

**DOI:** 10.1186/1475-925X-13-118

**Published:** 2014-08-19

**Authors:** Min-Hua Chen, Po-Chin Liang, Kai-Chun Chang, Jian-Yuan Huang, Yu-Ting Chang, Fuh-Yu Chang, Jau-Min Wong, Feng-Huei Lin

**Affiliations:** Institute of Biomedical Engineering, National Taiwan University, No.1, Sec.1, Jen-Ai Rd., Taipei, 100 Taiwan; Graduate Institute of Clinical Dentistry, School of Dentistry, National Taiwan University, Taipei, 10051 Taiwan; Department of Internal Medicine, National Taiwan University Hospital, Taipei, 10051 Taiwan; Department of Mechanical Engineering, National Taiwan University of Science and Technology, Taipei, 10607 Taiwan; Institute of Biomedical Engineering and Nanomedicine, National Health Research Institutes, Miaoli County, 35053 Taiwan

**Keywords:** Cholangiocarcinoma, Photodynamic therapy, Chemotherapy, Biliary drug-eluting stent, Electrospinning and Electrospraying

## Abstract

**Background:**

The combination of biliary stent with photodynamic and chemotherapy seemed to be a beneficial palliative treatment of unresectable cholangiocarcinoma. However, by intravenous delivery to the target tumor the distribution of the drug had its limitations and caused serious side effect on non-target organs. Therefore, in this study, we are going to develop a localized eluting stent, named PDT-chemo stent, covered with gemcitabine (GEM) and hematoporphyrin (HP).

**Methods:**

The prototype of PDT-chemo stent was made through electrospinning and electrospraying dual-processes with an electrical charge to cover the stent with a drug-storing membrane from polymer liquid. The design of prototype used PU as the material of the backing layer, and PCL/PEG blends in different molar ratio of 9:1 and of 1:4 were used in two drug-storing layers with GEM and HP loaded respectively.

**Results:**

The optical microscopy revealed that the backing layer was formed in fine fibers from electrospinning, while drug-storing layers, attributed to the droplets from electrospraying process. The covered membrane, the morphology of which was observed by scanning electron microscopy (SEM), covered the stent surface homogeneously without crack appearances. The GEM had almost 100% of electrosprayed efficiency than 70% HP loaded on the covered membrane due to the different solubility of drug in PEG/PCL blends. Drug release study confirmed the two-phased drug release pattern by regulating in different molar ratio of PEG/PCL blends polymer.

**Conclusions:**

The result proves that the PDT-chemo stent is composed of a first burst-releasing phase from HP and a later slow-releasing phase from GEM eluting. This two-phase of drug eluting stent may provide a new prospect of localized and controlled release treatment for cholangiocarcinoma disease.

## Background

Cholangiocarcinoma is the second most common hepatobiliary tumor, which is generally a locally invasive tumor that occludes the biliary tree and leads to cholangitis and liver failure. Until now, tumor resection has been the only potential cure for cholangiocarcinoma [1, 2]. Unfortunately, even with resection, the survival rate with five years can decrease to 11% at most and more than 50% of patients still remained at unresectable stage [3, 4]. Inoperable patients with advanced cholangiocarcinoma typically have obstructive cholestasis. So far, the primary standard method of treatment has been biliary stenting [5]. However, this treatment can prolong survival time slightly by providing temporary biliary drainage. Therefore, the secondary method of treatment is required to prolong the survival time by reducing tumor burden. Chemotherapy and radiotherapy are classical treatments but their results are also disappointing [3, 5].

Photodynamic therapy (PDT) is a new and promising treatment option, which contains a photosensitizer, light source, and oxygen [6]. The concept of PDT is based on a photosensitizer exposed to the specific wavelength of light, which can generate cytotoxic reactive oxygen species (ROS) to kill tumor cells [7]. Additionally, previous studies have shown that PDT could also inhibit the P-glycoprotein efflux of drug. A combination of PDT and chemotherapy can improve the accumulation of chemo-drug in tumor cells, and reduce the chemo-drug resistance from the P-glycoprotein efflux [8]. Another advantage of combination therapy with PDT and chemo-drug is the capability to induce antitumor immunity [9]. However, by intravenous delivery to target tumor, the distribution of the drug had its limitations and caused serious side effect on non-target organs. After receiving PDT treatment, patients have to stay indoors, away from bright light for 3 to 4 days to avoid the skin photosensitivity from the side effect [10].

In order to decrease the side effect during the treatment, the aim of this study is to develop a localized drug eluting stent, named PDT-chemo stent, by incorporating gemcitabine (GEM) with hematoporphyrin (HP) to cover the stent surface. Drug-eluting stent has been considered a method to maximize the drug concentration immediately on the localized tumor environment, while minimizing the non-target organs exposure [11, 12]. In clinical practice, this PDT-chemo stent could be inserted to the tumor area via endoscopic retrograde cholangiography [13], followed by the simultaneous specific light source from endoscopy to activate the photosensitizer for PDT. Meanwhile, the chemo-drug of GEM will be released continuously as the second step for chemotherapy. The multimodal function of PDT-chemo stent will not only aim to increase the accumulation of drug within the neoplastic tissue, but also decrease the side effect on non-target tissues.

**Figure 1:**
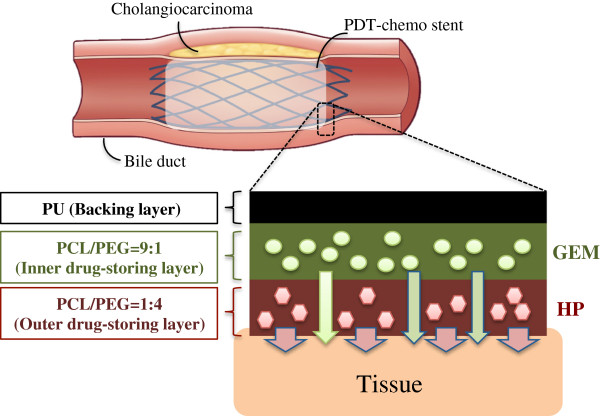
**Schematic illustration of the PDT-chemo stent as a biliary stent for cholangiocarcinoma treatment.** The covered membrane is composed of three layers with PU, PCL/PEG = 9:1 blends and PCL/PEG = 1:4 blends. PU was employed as the material of the backing layer, and PCL/PEG blends were used in drug-storing layers with GEM and HP loaded.

In the past decade, the stent with drug-incorporated covered membrane has been received increasing attention as drug-eluting stent due to its functions of providing mechanic support and releasing sufficient drug to prevent restenosis or treat malignant [12, 14, 15]. Historically, several techniques were developed and have been used to manufacture the covered membrane on stent surface. These diverse techniques include dip coating [12, 16], compression technique [14] and electrospinning [17, 18]. Concerned about the adherence and flexibility problems of the membrane covering the stent [19], we used the electrospinning technique. Electrospinning is the process with voltage to extrude polymer solution into fine fibers for the production of micro/nano-fiber-covered stents [20]. The electrospinning technique could support the covered membrane uniformly adhered to the stent and easily regulate the thickness according to the clinical needs [18, 19]. Therefore, we selected the electrospinning to construct the backing layer on 316 L stainless stent and used the electrospraying process to regulate GEM and HP on the covered membrane. To provide the flexibility of the membrane, polyurethane (PU), with sufficient elastic property, was used as the material of drug-free backing layer, which could effectively control majority of the inner drugs released to the tissue-contacting side and additional supporting force for the main drug layer [18]. Polycaprolactone (PCL) and Polyethylene glycol (PEG) blends in different molar ratio were selected as two drug-storing layers to control the drug-releasing rate. In the ratio of 1:4, PCL/PEG blends were used as the outer releasing layer with HP loaded, while in the ratio of 9:1, PCL/PEG blends were used as the inner releasing layer with GEM loaded (Figure 1). To our knowledge, this is the first study to show that the biliary stent could be accompanied with PDT and chemotherapy for localized cholangiocarcinoma treatment.

## Methods

### Materials

Polyurethane (PU), Polycaprolactone (PCL, Mw = 80,000), Polyethylene glycol (PEG, Mw = 20,000), Tetrahydrofuran (THF), 1,1,1,3,3,3-Hexafluro-2-propanel (HFIP) and Hematoporphyrin (HP) were purchased from Sigma-Aldrich (St Louis, MO). Gemcitabine (GEM) of clinical grade was supplied by National Taiwan University Hospital. All other chemicals were of analytical grade and used as received.

### Preparation of PDT-chemo stent

The PDT-chemo stent, consisting of tri-layers of membranes, was made by electrospinning and electrospraying dual-processes. The metallic stent was provided from the laboratory of Dr. Fuh-Yu Chang in National Taiwan University of Science and Technology, and the femtosecond laser was used to carve the 316 L stainless metallic tube. The unit of electrospinning contained a high-voltage power supply, a motor to rotate the stent, a syringe pump, and a 19G-needle that was connected by a tube to a syringe. The metallic stent rotated by a motor was horizontally placed 15 cm away from the needle. The solution of PU in HFIP (10 m/v%) was used for electrospinning process, while PCL/PEG blends were mixed in HFIP/THF (1:1) solution for electrospraying process. Both PU and PCL/PEG blends solution were extruded from the syringe at a rate of 5 μL/min. The backing layer of PU was first electrospun with voltage 14 kV, and was followed by the drug-storing layer of PCL/PEG blends elecrosprayed with higher voltage 22 kV. Herein, the drug-storing layer of PCL/PEG blends in molar ratio of 9:1 was loaded with GEM covering the backing layer, followed by the HP coating on the top in PCL/PEG molar ratio of 1:4 (Figure 1). The extruded polymer from the syringe of electrspinning/electrospraying was collected for only a short period time on cover glass for optical microscopy (OM, Leica, Germany). Samples collected covered stent were prepared by coating with thin gold film by sputtering PVD and visualized by scanning electron microscopy (SEM, JSM-7000 F, Japan) operated at 15 KV.

### Drug electrosprayed efficiency

To further confirm the electrsprayed efficiency of loading drug in state of covered membrane, the membrane was collected from the stent and absolutely dissolved in dimethyl sulfoxide (DMSO) solution. After that, high-performance liquid chromatography (HPLC, Waters e2695, USA) and ultraviolet–visible spectroscopy (UV/vis, JASCO V-550, USA) were used to examine the extruded drugs of GEM and HP from the covered membrane respectively.

### Drug release

The covered membrane was incubated in a sealed glass bottle with 0.5 ml phosphate-buffered saline (PBS) as the releasing medium. The bottle was placed in a shaking incubator at 37°C at a shaking speed of 50 rpm. At the predetermined time, 0.5 ml sample was withdrawn and replaced with the same volume of fresh medium. Residual concentration of drug in the membrane was counted by dissolving the membrane in DMSO solution as the eluting medium. Samples were collected and analyzed under the UV–vis spectrometer and HPLC. The morphology of membrane after 72 h of release was assessed by SEM imaging. The values were presented as mean ± standard error (STD) in triplicate. Statistical analysis was performed using the analysis student’s t-test. Values of p < 0.05 was considered being statistically significant.

## Results and discussion

### Prototype of PDT-chemo stent

To make the stent with covered membrane more uniformly adhered to the surface and easily regulate the thickness according to the clinical needs, electrospinning/electrospraying has been regarded as the appropriate means for demonstrating the PDT-chemo stent [18, 19]. The covered stent can be manufactured by several electrospinning methods: post-spinning modification, drug/polymer blends, emulsion electrospinning and core-shell electrospinning [21]. Drug/polymer blends technique could easily mix the drug with polymer directly and form a layer of membrane to achieve sustained drug release. Therefore, in this study, the prototype of PDT-chemo stent was constructed by drug/polymer blends technique via electrospinning and electrospraying dual-processes. The backing layer was electrospun first from PU polymer solution, followed by the electrospraying process from PCL/PEG blends solution with drug loaded. Electrospinning is the process with voltage to extrude polymer solution into fine fibers for the production of fine-fibers-covered stents. In our case, fine fibers could support the superior mechanical properties of the membrane and be introduced as the backing layer for effectively controlling majority of the inner drugs released to the surrounding tissue. During electrospinning, the organic solvent, which could be toxic to cells, will be completely evaporated due to its high volatility [22]. Electrospraying has similar preparation process to electrospinning but is usually used with higher voltage and lower polymer density, which makes the polymer solution more easily broken up into droplets [23, 24]. The concept of electrospraying process was used to increase layer-to-layer adhesion, which could avoid drug-storing layer cracking and separating from backing layer during the stent expending.

The covered membrane is composed of three layers with PU, PCL/PEG = 9:1 blends and PCL/PEG = 1:4 blends. PU was employed as the material of the backing layer, while PCL/PEG blends were used in drug-storing layers with GEM and HP loaded (Figure 1). Several materials (eg. Silicone, PTFE, and PU) have been approved by US Food and Drug Administration (FDA) as the covered membrane on the stent [13]. Silicone was reported to cause the acute inflammation to the surrounding tissue [25]. The PTFE, however, could not be dissolved into any solvent [26]. Therefore, as the material of the backing layer in this study, PU has been found able to be formed sufficiently thin and flat on the metallic stent by electrospinning process and with elastic properties to allow the covered stent to be homogeneously expanded [18]. Additionally, PU membrane has been proved to prevent the tumor ingrowth effectively and to reduce the occlusion rate of expandable metal stent in patients with malignant biliary obstruction [15]. The distinct biodegradable properties of PCL and PEG blending were regarded as an approach to controlling the drug-releasing rate in different blending ratio. The selection of PCL is due to its good biocompatibility, drug permeability and relatively slow degradation rate [27, 28]. The hydrophilic PEG was selected to play a role in resulting in regulating the drug-releasing rate, due to its easily acting on aqueous solution [14, 29].

**Figure 2:**
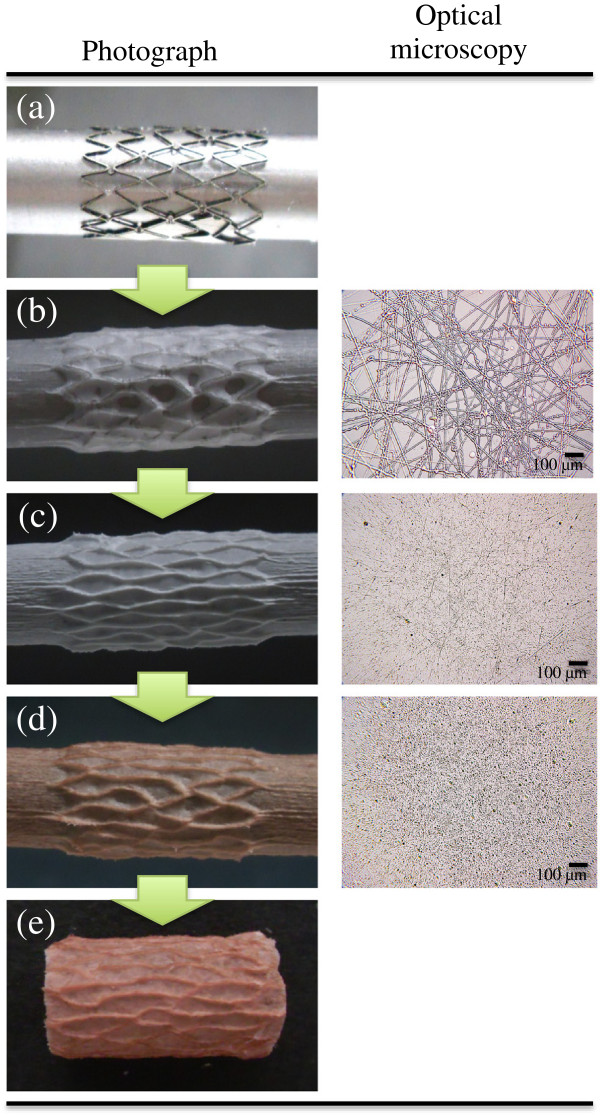
**The photography and microscopic imaging of PDT-chemo stent.** The prototype of PDT-chemo stent in each step of electrospun/electrosprayed process is presented under the photography and optical microscopic imaging. **(a)** 316 L stainless bare stent; **(b)** covered with PU backing layer; **(c)** covered with inner drug-storing layer by PCL/PEG = 9:1 with GEM loaded; **(d)** covered with outer drug-storing layer by PCL/PEG = 1:4 with HP loaded; **(e)** the prototype of PDT-chemo stent collected from cylindrical collector. Optical microscopy shows the extruded polymer from the syringe of electrspinning/electrospraying processes.

**Figure 3:**
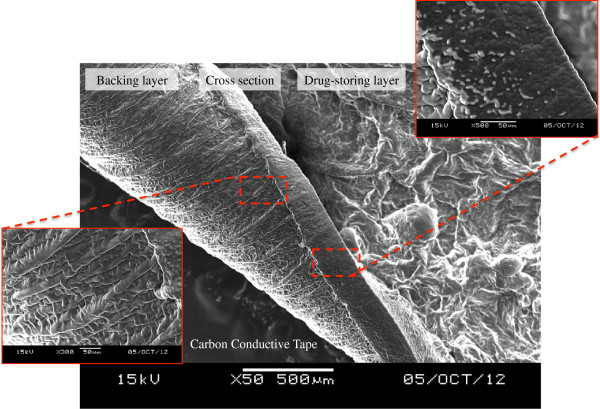
**SEM imaging of the covered membrane.** The surface morphology and cross section of the covered membrane from PDT-chemo stent was observed by SEM imaging, which showed that the membrane was constructed of two sides with the backing layer and drug-storing layer.

The prototype of PDT-chemo stent was imaged by photography and optical microscopy, which proved that the membrane was homogeneously covered on the stent surface in each electrospun process (Figure 2). The optical microscopy (Figure 2b) revealed that PU was formed in fine fibers with width around 5 to 10 μm; thus PCL/PEG blends solution was favorable for generating submicron droplets (Figure 2c, d). The photograph of membrane appeared brown (Figure 2d) due to the homogeneously dispersed of HP coated on the top. Finally, the prototype of PDT-chemo stent could be easily removed from the cylindrical collector without any surface damage (Figure 2e). The surface morphology and cross section of the film was further investigated by SEM imaging (Figure 3), which showed that the membrane was constructed of two sides with the backing layer and drug-storing layer. The architecture of the backing layer was constructed of fine fibers in networks structure, while drug-storing layer, attributed to the droplets from electrospraying process was coated on the backing layer roughly. The cross section of the membrane was smoothly with width in range from 170 to 190 μm. The thickness of the membrane could be optimized for the most favorable thickness according to the clinical needs via regulating time during the electrospun process.

**Figure 4:**
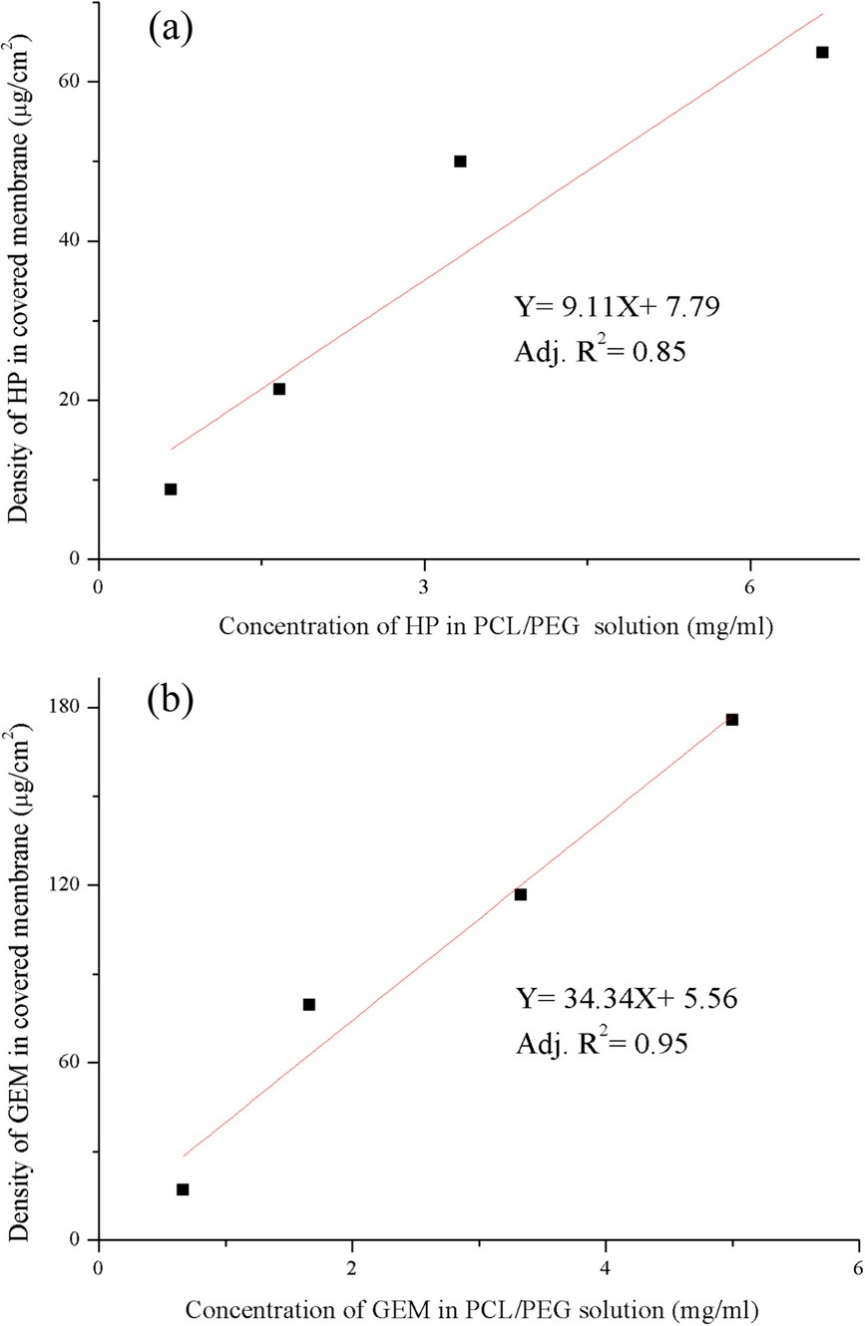
**The electrosprayed amount of the drug on the covered membrane.** The density of **(a)** HP and **(b)** GEM coated on the covered membrane is illustrated on the vertical axis of this graph, while the drug concentration of HP and GEM in PCL/PEG blends solution respectively is illustrated on the horizontal axis.

To further confirm the electrosprayed amount of the drug on the covered membrane, the membrane removed from the stent was completely dissolved in DMSO solution for further analysis. The results revealed that, with the increasing concentration of the HP in PEG/PCL solution from 0.67 mg/ml to 6.67 mg/ml, the density of the HP from the electrosprayed membrane increased gradually from 8.72 μg/cm^2^ to 63.59 μg/cm^2^ at most, which was around 0.7 fold to the HP containing solution, as seemed to demonstrate 70% of electrosprayed efficiency (Figure 4a). By the same method, GEM had almost 100% electrosprayed efficiency (Figure 4b) and had a tendency of increasing electrosprayed efficiency by mixing with PCL/PEG blends due to its high hydrophilicity [30]. The corresponding results of the concentration of the drug in the solution and the density of the covered membrane could be an useful information to further imitate a clinical dose regimen for cholangiocarcinoma treatment.

### Effect of drug release

**Figure 5:**
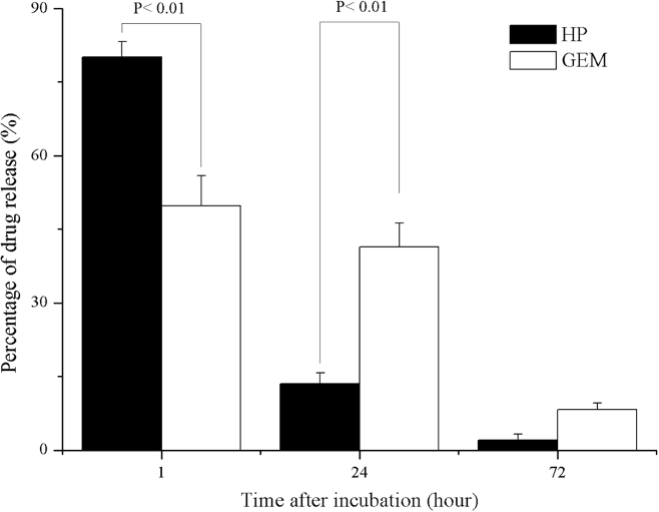
**The profile of drug release.** Release of HP and GEM from covered membrane at each predetermined time in PBS solution (mean ± STD, n = 3). Values of p < 0.01 is considered being statistically significant.

As illustrated in Figure 1, this PDT-chmo stent was designed to obtain a two-phased drug release pattern, which is composed of a first burst-releasing phase and a later slow-releasing phase with combination therapy for cholangiocarcinoma treatment. Hydrophilic PEG was used as a regulator of drug release from PCL/PEG blends [31]. Figure 5 displays the drug releasing profiles from the covered membrane. At each releasing time, the cumulative amounts of HP from PCL/PEG = 1:4 membrane, released in the first hour (P < 0.01 relative to GEM), had a high initial burst 80% and reached maximal cumulative release of nearly 98% within 24 h. Whereas, compared to HP in the first hour, GEM within PCL/PEG = 9:1 membrane showed relatively slow drug release in the first hour (50%). The significant difference of releasing kinetics between HP and GEM was observed within 24 h, as indicated drug-releasing rate can be regulated within 24 h by adjusting PEG and PCL compositions, then both drugs complete releasing occurred during the time span between 24 h and 72 h. The mechanism of drug release was reported by Liu et al. [32] and Lei et al. [14] that PEG acted as a pore former in PCL/PEG blends, where the releasing rate from co-localization of protein/drug and blends were proportional to PEG content. The hydrophilic PEG in PCL/PEG blends easily acted on aqueous solution, which resulted in the formation of swollen structure and subsequently increased the drug-releasing rate, as indicated the kinetics of drug releasing was mainly due to the degradation of the PCL/PEG blends [14]. Although the slow releasing rate of GEM may not exclude the possibility of outer drug-storing layer (PCL/PEG = 1:4) to delay parts of GEM release, we consider that PCL/PEG = 1:4 membrane with high concentration of hydrophilic PEG polymer will interact fast with aqueous solution, leading the membrane to degrade quickly within the first few minutes [14]. Therefore, we suggest PCL and PEG with different molar ratio for controlling the polymer degradation rate be the major factor to regulate drugs releasing rate. In order to better meet the needs of clinical application, the thickness of covered membrane and membrane composition could be further easily improved according to our requirements by electrospinning/electrospraying technique [18, 19].

**Figure 6:**
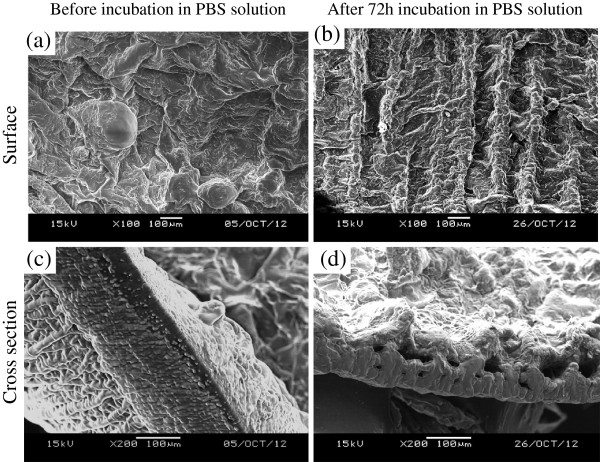
**SEM imaging of eluted covered membrane.** After 72 h drug eluting in PBS solution, the surface morphology **(a, b)** and cross section **(c, d)** of the covered membrane were observed by SEM imaging.

After drug eluting in PBS solution, the surface morphology of the covered membrane was investigated by SEM imaging (Figure 6). Generally, fine fiber architecture of the backing layer was covered with roughness of the drug-storing layer (Figure 6a). After 72 h of drug eluting, rough surface of the drug-storing layer was converted to alignment (Figure 6b). This orderly structure indicates the degradation of PCL/PEG blends from the surface, and only PU backing layer and partial PCL/PEG blends left on the layer can be observed. The thickness of the cross section was reduced from around 180 μm (Figure 6c) to 120 μm (Figure 6d). The SEM imaging further confirmed the drug release kinetics was mainly because of the degradation of PCL/PEG membrane, not due to the diffusion or permeation of drugs through the membrane.

Herein, GEM is one of the first line chemo-drugs in the treatment of advanced cholangiocarcinoma, which is a prodrug belonging to an analog of deoxycytidine. Once GEM is transported into the cell, it will be phosphorylated to an active form to inhibit DNA synthesis [33]. Therefore, low initial burst of GEM may help to prevent undesired toxicity associated with high concentration of GEM, and the burst release of HP can provide a simultaneous treatment for PDT, triggered by the light source from endoscopy when the stent is localized in bile duct. Overall, the prototype of PDT-chemo stent has demonstrated the proof of concept of localized combination therapy for cholangiocarcinoma. Based on the theory, the drug-releasing rate could be further regulated by changing the initial electrospraying blend polymer solution, concentration, structure and type of fibers and the amount of additives for the clinical needs [34, 35].

## Conclusions

In preliminary study, we have successfully developed a prototype of tri-layered covered stent with PDT and chemotherapy. This PDT-chemo stent was prepared by electrospinning and electrospraying dual-processes. The membrane is composed of PU backing layer as the base and PCL/PEG drug-storing layers with GEM and HP on the top. The mixing of drugs with different PCL and PEG composition demonstrated an effective strategy for regulating the drugs release from the membrane. The release study has confirmed a two-phased drug release pattern, which provides a proof of concept for the hypothesis that the PDT-chemo stent is composed of a first burst-releasing phase from HP and a later slow-releasing phase from GEM eluting. This two-phase of drug eluting stent may provide a new prospective of localized controlled release treatment for cholangiocarcinoma disease.
